# Eyesight

**Published:** 1863-05

**Authors:** 


### EYESIGHT.

IN Health Tracts 5, 15, and 61 we have said a number of very sensible things about preserving the eyes, and putting far off the evil day of 'specs.' Some saucy exchange, not having the fear of our displeasure properly before its eyes, has audaciously essayed to poke fun at us in the manner and form to wit:

"ADVICE TO PERSONS POSSESSING EYES.

AFTER HALL'S JOURNAL OF HEALTH.

"The eye is probably the best apparatus ever constructed for seeing things, always excepting opera and quizzing glasses. Without it, merchants would be everlastingly doomed to 'go it blind,' as they have been doing ever since the panic of 1857; and man could not extend his vision to surrounding objects. The eye is subject to not less than six hundred diseases, the most prevalent among which is the 'pink-eye,' common only to potatoes, the human family, and poodle-dogs of delicate constitutions. The professions are unanimously of the opinion that it is incurable. As man is very rarely favored with a second pair of eyes, it is but common prudence to take care of the brace furnished him at the time of setting out to seek his fortune; and therefore the following rules will be found serviceable.

"Reading by a candle, unless it is lighted, is very hurtful, and should never be indulged in except by daylight. Absent-minded persons, please notice.

"Intoxicated persons should not attempt to read, as their staggering causes a continual and painful change of the focus of the eye.

"The practice of reading when going down-town is hurtful; if walking, you are liable to rudely encounter a school-girl, also reading; and if in a 'stage,' your attention may inconvenience the lady occupants, who expect you to pass up their pennies or steady their baskets.

"Never attempt to look at the sun, unless you have glass eyes; and when you patronize street-telescopes, do not grumble when paying for an 'interesting view' of Sol, and say, 'you can't see it.' It speaks bad for your eyes.

"Do not look at the moon, as the man in it might consider it impertinent, and being a lunatic, might cause you trouble.

"The glare of the sun on water is very bad for the eyes, and for this reason a person should always drink something else during the daytime.

"'Seeing stars' and prize-fighting are hurtful to the eye.

"If compelled to fight, avoid black eyes; they greatly discourage the natural sight, and are the reverse of ornamental.

"'Keeping your eye peeled' is not a literal expression; it should be taken figuratively, as the 'peeling' process is bad for the optic.

"As any sudden change from darkness to a bright light is injurious to the eye, all fireworks should be set off in the daytime, and Barnum's calcium light should be extinguished.

"Never attempt to read by the light of a burning building, as the fire may be put out before you finish the story; besides, you would be in danger of getting hit by a brick, or run down by one of 'Eighty's boys.'

"In looking at minute objects the eye should be occasionally relieved by the sight of a 'big thing.' For instance, when looking down the throat of a mosquito to see where your blood has gone, have Barnum's hippopotamus at hand, with open mouth, to give variety to the view, thus resting the eye.

"On arising in the morning, if the eyes are matted together, it is very hurtful to have a fire-engine to play into them, and a person should never wash his eyes of a morning in gin and bitters, as the 'stoughton' is very apt to discolor the optic nerve. The proper and most agreeable method of performing this feat is, soak the optics not to exceed two hours in warm soap-suds, and then pry the lids open with an oyster-knife. The cause of the adhesion can then be removed by an application of sand-paper and elbow-grease.

"Never bathe the eyes in cold water, it is apt to give them the cramp, and has been known to produce gout in the retina.

"Ordinarily, spectacles should be worn by elderly people only, though many young gentlemen can see very well through a 'pair of glasses.' They are, however, extremely apt to affect the tongue and the breath.

"Persons with long sight should look at an unpaid tailor's or milliner's bill by holding it close to the eye, as they can then truthfully declare that they 'can't see it.'

"Some individuals are troubled by the rapid growth of their eye-lashes, (winkers is the professional term,) which is caused by an undue proportion of bear's oil in the fatty substance of the optic. If the lashes become too long, do not cut them with a mowing-machine; it is both unnecessary and expensive. Besides, it is attended with danger—the books containing a number of cases where the sight has been permanently injured by running a number of the teeth of a mower into the eye. The proper mode of abbreviation is, to trim them carefully with an apple-paring machine.

"If the eyes are not of the same color, the owner should not attempt to establish a uniformity by the use of hair-dye or wash, unless he has consulted a fortune-teller on the subject. Even then the risk is great, and no regular practitioner should attempt the operation unless paid in advance.

"Near-sightedness is caused by the inability of certain persons to see objects at any great distance; it can be cured by lengthening the distance at which objects are visible. If the eyes experience an itching sensation, never rub them with the finger; the saline matter in the insensible perspiration making the optics more irritable. Draw a currycomb gently over the eye, from the nose outward, avoiding that prominent organ, especially if a wax one.

"Double-sight is very dangerous, and persons should be 'treated' promptly when thus afflicted.

"By observing the above, eyes will not give out until the vision begins to fail. ZEBEDEE SQUELCH, M.D."

We greatly regret that Professor Squelch did not send us a lock of his hair with his basket of fun, as we should have taken pains to have it placed in a promiscuous position in "Barnum's Music," as our little Alice used to say, with his name and residence attached thereto, and thus have aided in giving him a name and a fame second only to that of Margulies of the Lafarge, whose skill and ability as a scientific oculist, is confessedly superior to that of any other man in this country. Meanwhile we beg to assure our friend Zebedee, that he is the esteemed object of our very considerable consideration.

